# Diet and Carbohydrate Food Knowledge of Multi-Ethnic Women: A Comparative Analysis of Pregnant Women with and without Gestational Diabetes Mellitus

**DOI:** 10.1371/journal.pone.0073486

**Published:** 2013-09-12

**Authors:** Habiba I. Ali, Amjad H. Jarrar, Mohamed El Sadig, Karin B. Yeatts

**Affiliations:** 1 Department of Nutrition and Health, College of Food and Agriculture, United Arab Emirates University, Al Ain, UAE; 2 Department of Community Medicine, College of Medicine and Health Sciences, United Arab Emirates University, Al Ain, UAE; 3 Department of Epidemiology, Gillings School of Global Public Health, University of North Carolina at Chapel Hill, North Carolina, United States of America; Iran University of Medical Sciences, Iran (Republic of islamic)

## Abstract

**Background:**

Diet therapy is the cornerstone for the management of gestational diabetes mellitus (GDM). Carbohydrate is the primary nutrient affecting postprandial blood glucose levels. Hence, knowledge of food containing carbohydrates can assist women with GDM optimize glycemic control. Despite that, there is a paucity of research on carbohydrate-related knowledge of women with GDM. The United Arab Emirates (UAE) has one of the highest prevalence of diabetes (19.2%) in the world. This study compared diet and knowledge of carbohydrate-containing foods among pregnant women with and without GDM in the UAE.

**Methods:**

The sample consisted of multi-ethnic women with GDM (n = 94) and a control group of healthy pregnant women (n = 90) attending prenatal clinics in three hospitals in Al Ain, UAE. Data were collected using a questionnaire and a 24-hour recall. Knowledge of food sources of carbohydrate, dietary patterns, and nutrient intakes of the two groups were compared.

**Results:**

There were no significant differences in the mean knowledge score of food sources of carbohydrate between women with GDM and that of pregnant women without GDM. Similarly, there were no significant differences in energy and nutrient intakes between the two groups with the exception of percent energy from protein. Women with GDM reported significantly lower intake of fruits and fruit juices (P = 0.012) and higher consumption of milk and yogurt (P = 0.004) compared to that of women without GDM. Twenty-two percent of women with GDM indicated they never visited a dietitian for counseling while 65% reported they visited a dietitian only once or twice during the pregnancy. Predictors of carbohydrate knowledge score were perceived knowledge of diet and GDM and parity among women with GDM and parity and educational level among those without GDM.

**Conclusion:**

The results of the study highlight the urgent need to provide nutrition education for women with GDM in the UAE.

## Introduction

Gestational diabetes mellitus (GDM) is defined as any degree of glucose intolerance with onset or first recognition during pregnancy [1], [2]. Risk factors for the development of GDM include advanced maternal age, family history of type 2 diabetes mellitus, obesity before pregnancy, previous macrosomic infant (birth weight >4000 g), and excess maternal weight gain in current pregnancy [3]. Gestational diabetes is associated with specific ethnicities, including Hispanic, black, Native American, South or East Asian, Middle Eastern, Chinese, Pacific Islander, and Indigenous Australian [4]. Gestational diabetes can lead to significant morbidities for mother and baby, both perinatally and in the long term [4]. Higher rates of stillbirth, poly-hydramnios, gestational hypertension, macrosomia, and caesarean have been reported among women with gestational diabetes [5]. Moreover, although gestational diabetes usually resolves following birth, it is known to confer an increased risk for recurrent GDM in subsequent pregnancies, type 2 diabetes mellitus, and cardiovascular risk factors later in life [6], [7]. The risk of developing type 2 diabetes in women with history of GDM may vary from 17 to 63% within 5–16 years after pregnancy, depending on ethnicity and the method used for screening [8], [9].

Medical nutrition therapy is an integral component of diabetes prevention and management and is recognized as an essential component of an overall healthy lifestyle [10], [11]. Moreover, there is strong evidence supporting dietary modifications and changes in lifestyle for the treatment of GDM [10], [12]–[15]. Diet therapy is recognized as the cornerstone for the management of gestational diabetes mellitus [16]. Nutrition counseling opportunities can optimize maternal and fetal outcomes [14], [15]. Moreover, nutrition counseling during pregnancy can promote adoption of healthy dietary patterns and reduction of future GDM related risk factors, including type 2 diabetes [10]. In fact, medical therapy practice guidelines recommend a minimum of 3 visits with a dietitian [16]. An optimal diet for a pregnant woman with GDM provides adequate nutrition for fetal growth and maternal health while minimizing hyperglycemia and excessive weight gain. Nutrition counseling in women with GDM resulted in decreased intake of energy and saturated fatty acids [17], decreased weight gain during pregnancy in obese women [18], and decreased maternal weight gain and fasting serum glucose levels [19].

Carbohydrate–containing foods have the greatest impact on postprandial blood glucose levels. Nevertheless, carbohydrate –containing foods can be important sources of energy, vitamins, minerals, and fiber. It is recommended that women with GDM to consume at least 175 g of carbohydrate per day according to the Institute of Medicine Dietary Reference Intakes (DRIs) [20]. On the other hand, excessive intakes of carbohydrate, especially in diet-controlled GDM can lead to hyperglycemia [21]. For example, diets containing a high proportion(>55%) of energy from carbohydrates had been shown to lead to higher levels of postprandial blood glucose levels in gestational diabetes [22].

Since gestational diabetes is a form of glucose intolerance, it is critical that women with GDM acquire knowledge about foods that contain carbohydrate to facilitate food choices that will not result in hyperglycemia. The American Diabetes Association recommends monitoring carbohydrate either by carbohydrate counting or experience-based estimation as a key strategy in achieving glycemic control [23]. Thus, counting of carbohydrate content in food is often used as a method for meal planning for people with diabetes. The impact of carbohydrate on blood glucose levels can be affected by the total amount of carbohydrate and the type of carbohydrate [16]. Although the research is not conclusive [14], studies have reported the beneficial effects of using low glycemic index (GI) foods in the management of GDM [24]–[28].

A major focus of medical nutrition therapy for GDM is modifying both the carbohydrate content and type of the meal plan to achieve and maintain normoglycemia while ensuring that nutrient needs are met [29]. Distributing the carbohydrate intake throughout the day in three small-to moderate meals and two to four snacks is recommended [30]. Moreover carbohydrate is less tolerated during breakfast compared to other meals [10]. However, the adoption of these guidelines by women with GDM requires knowledge of food sources of carbohydrate.

Despite its importance, little research has been conducted in the UAE and elsewhere to determine knowledge of foods that contain carbohydrate among women with GDM. To our knowledge, only one study involving multi-ethnic women in Australia included carbohydrate food in assessing the nutrition knowledge level of women with gestational diabetes [31]. The United Arab Emirates (UAE) is a multi-ethnic community with one of the highest prevalence (19.2%) of diabetes in the world [32] and has a high prevalence of gestational diabetes, multiparity [33]–[35], and obesity [36]. Using 6 internationally well-accepted GDM diagnosis criteria, the prevalence of GDM in the UAE ranged from 7.9% to 24.9% [34]. To the best of our knowledge, currently there are no studies on food choices or nutrition knowledge of women with GDM in the UAE. Such research is critical in providing targeted nutrition advice to women with GDM. Therefore, the objective of this study was to explore the knowledge related to food sources of carbohydrate and energy and nutrient intakes of women with GDM in the UAE and to compare with those of pregnant women without GDM.

## Methods

### Ethics Statement

The protocol of the study was approved by the Research Ethics Committee of the United Arab Emirates University and by the authorities from the three hospitals where the study was conducted. All participants gave verbal and written consent.

### Design and Participants

This was a cross-sectional comparative study of women with and without GDM. We recruited a sample of multi-ethnic women with GDM (n = 94) and a control group of healthy pregnant women (n = 90) attending prenatal clinics of three major hospitals in Al Ain District, United Arab Emirates (UAE). Data were collected during the period of March and April 2009. The sample frame consisted of all pregnant women presenting to the prenatal clinics in the three selected hospitals in two fixed days a week, throughout the study period. As part of a universal screening program, pregnant women attending the prenatal clinics of the three hospitals undergo a 75 g 2-h oral glucose tolerance test (OGTT) scheduled between 24 and 28 weeks gestation.

Treating doctors identified the eligible women (i.e. women diagnosed with GDM and pregnant women without GDM in their third trimester of pregnancy) before referring them to the study team for consideration. The inclusion criteria were: (1) Pregnant women with or without GDM in their third trimester; (2) attending one of the three participating prenatal care clinics; and (3) able to communicate fluently either in Arabic or English language. Those in their first and second trimester of pregnancy as well as those with pre-existing diabetes were excluded from the study. The study team approached 214 pregnant women who attended the 3 participating prenatal clinics during the period of the study. Of those invited to participate, 30 women (14 with GDM and 16 women without GDM) refused to take part in the study. Thus, the refusal rate for the study was 14% and the major reason for refusal was time constraints. Women who gave consent were recruited for the study and were interviewed once until the target date of end of data collection was reached.

### Data Collection and Analysis

It was not possible to find an appropriate questionnaire to assess carbohydrate –food knowledge of women with GDM. Thus, the research team consisting of a diabetes educator, public health specialists, and a nutritionist developed the questionnaire that was used for the study. The questionnaire which was originally developed in English, was translated into Arabic, then back translated into English, and reviewed independently by two bilingual (Arabic & English) researchers to ensure that the translations were accurate, reflected local terminology, and appropriate for use in the field.

The questionnaire covered questions relating to demographic, medical history, pre-pregnancy weight, and meal and snack frequency during pregnancy. Leisure and housework-related physical activity levels during the week preceding the interview were also obtained from each participant. Women with GDM were asked, “How much knowledge do you have about diet and GDM?” with the following three possible responses: “A lot of knowledge”, “some knowledge”, and “little knowledge”. They were also asked whether they self-monitor their blood glucose levels.

Participants were asked about consumption of fruits, vegetables, and milk on a typical day during the week preceding the interview. The following question was asked, “During the past week, on an average how much of the following foods did you eat/drink per day?” In order to quantify their food intake, the following serving size options were given “less than 1 cup/d, 1–2 cups/d and 3 cups or more per/d.” This question format is based on semi-quantitative food frequency technique in published literature [37]–[39]. Serving sizes for individual food groups were defined in terms of the MyPyramid servings [40]. Total servings from the fruit group were obtained by summing up fruit juices and fresh fruits (2 medium fruits were counted as 1 cup) according to the MyPyramid Food Guide portion sizes. During the interview participants were shown a measuring cup to assist them in food portion estimations.

Knowledge of carbohydrate-containing foods was assessed using a questionnaire containing a list of 14 food items that are commonly consumed in the UAE. This list was previously used to assess the carbohydrate-food knowledge of adults with type 2 diabetes in the UAE and Oman [41]. Moreover, due to the fact that the food items included in the questionnaire are commonly consumed in the UAE, face validity was used to ensure the validity of the food items in the questionnaire. Participants were asked whether a specific food item, for example, rice, can increase blood glucose levels. The possible answers were: “Yes”, “No”, and “Refused”. Each correct response was assigned a value of 1 and an incorrect response was calculated as 0, with a possible maximum score of 14. Food items that normally contain more than 5 g of carbohydrate per serving were classified as having the potential to increase blood sugar levels (carbohydrate-containing food) [42].

The overall questionnaire was pilot-tested with 10 pregnant women (6 with GDM and 4 without GDM). The pre-test revealed the need for modifications of few of the questions which needed further simplification for respondents. Accordingly, the research team revised the questionnaire after careful consultations to ensure that the wording of each question will be easily understood by the respondents.

A single 24-hour food recall was used to assess energy and nutrient intakes. Trained interviewers conducted the dietary interviews, using food models and common household measures (spoons and cups) to assist participants in estimating food portions consumed. The 24-hour recall energy and nutrient analysis was performed using ESHA Food Processor SQL, software, v. 10.4 [43]. The ESHA software contains food composition database of more than 35,000 food items with data from more than 1500 sources, including the latest US Department of Agriculture Nutrient Database for Standard Reference, items from the US individual consumption survey databases, manufacturer’s data, data from fast food companies, and data from literature sources. Currently UAE does not have its food composition database and the USDA database was chosen for the analysis of energy and nutrient intakes for a number of reasons. The UAE depends mainly on food imports from all around the world; the USA is a key source. Moreover, the USDA nutrient database is considered one of the most comprehensive and up-to-date food composition databases. Finally, previous research conducted in the UAE [37], [44], [45] and in other countries in the Arab Gulf region [46], including development of a semi-quantitative food frequency questionnaire for the UAE and Kuwait [37] used the USDA food composition database. For the composite dishes and other food items not included in the ESHA database, the individual ingredients were entered into ESHA to determine the energy and nutrient content. Level of knowledge of food sources of carbohydrate, nutrient intakes, and dietary patterns of the women with GDM were then compared with those of the healthy pregnant women.

### Statistical Analysis

The data was coded and analysed using the Statistical Package for Social Sciences (SPSS) software (version 20). Descriptive analysis, using standard statistical methods was performed. Inferential statistical methods including the Chi-square tests and/or Fisher’s exact test and independent t-tests were used to ascertain associations between variables. Multiple, step-wise regression analysis was used to assess potential predictors of diet and carbohydrate-food knowledge score among GDM and non-GDM women. For the GDM group the list of potential predictors included in the model were age, educational level, parity, nationality, previous GDM, and level of participants’ knowledge on diet and GDM. The same predictors, except for previous GDM and level of participants’ knowledge about diet and GDM were used for the non-GDM group. P≤0.05 was considered the cutoff value for statistical significance.

## Results

### Demographic and Lifestyle Characteristics

Demographic and lifestyle characteristics of the participants are presented in Table 1. The major ethnic groups of the study population were UAE and expatriate Arabs (87%). Non-Arabs were mainly Indians. For women with GDM, 63% were UAE citizens, 24% were Arabs of other nationalities and the rest were non-Arabs (Table 1). In contrast, for the non-GDM women, 50% were UAE citizens, 41% were Arabs of other nationalities and the rest were non-Arabs. The mean age for women with GDM was 31.1**±**4.9 years **(**mean ± SD) compared to 27.5±6.0 for non-GDM women. Nearly 27% of the women with GDM had less than high school education compared to 16% of those without GDM. Slightly more than half (53%) of women without GDM had college education (Table 1). Nearly 48% of the women with GDM had a prior history of GDM, 40% had family history of GDM, and two-thirds of them had a family history for type 2 DM. Only 4.3% of the women with GDM were managed with insulin. Furthermore, 38% of women with GDM did not perform self-monitoring of blood glucose (Table 1). A comparison of the two groups showed that women with GDM in the study were older (P<0.0001) and had significantly higher pre-pregnancy BMI (29.6 vs. 26.9, respectively; P = 0.009) and parity (P = 0.004**)**. Similarly, known risk factors of GDM were found to be higher among women with GDM such as GDM in a previous pregnancy (47.9% vs. 3.4%, P<0.0001), family history of GDM (39.8% vs. 15.6%, respectively, P<0.0001), and family history of type 2 diabetes (65.9 vs. 26.7%, respectively, P<0.0001). There was no statistically significant difference in the usual physical activity levels reported by the women with GDM and those without GDM (Table 1).

**Table 1:** Demographic and lifestyle characteristics of pregnant women with and without GDM in Al-Ain District.

Characteristics	GDM (n = 94)		Non GDM (n = 90)		
	N (%)	Mean ± SD	N (%)	Mean ± SD	*P* Value*
Age in years		31.1±4.9		27.5±6.0	<0.0001^a^
Height (cm)		159.2±6.5		160.3±6.4	0.80^a^
Pre-pregnancy weight (kg)		71.5±19.2		64.1±15.8	0.005^a^
Pre-pregnancy BMI (kg/m^2^)		29.6±7.2		26.9±6.2	0.009^a^
**Nationality**					0.053^b^
UAE	59 (62.8)		45 (50.0)		
Non-UAE Arabs	23 (24.4)		37 (41.1)		
Others	12 (12.8)		8 (8.9)		
**Educational level**					0.045^b^
Less than high school	25 (26.6)		14 (15.6)		
High school diploma	35 (32.7)		28 (31.1)		
College or more	34 (36.2)		48 (53.3)		
**Parity**					0.004^b^
None	9 (10.2)		26 (29.2)		
1	10 (11.4)		12 (13.5)		
≥2	69 (78.4)		51 (57.3)		
GDM in previous pregnancy	45 (47.9)		3 (3.4)		<0.0001^b^
Family history of GDM	37 (39.8)		14 (15.6)		<0.0001^b^
Family history of type 2 diabetes	60 (65.9)		24 (26.7)		<0.0001^b^
**Treatment for GDM**					
Insulin	4 (4.3)				
Diet & exercise	90 (95.7)		–		
**SMBG** **					
Yes	58 (61.7)		–		
No	36 (38.3)				
**Leisure Physical Activity during the previous week**					0.693^b^
Yes	57 (60.6)		52 (57.8)		
No	37 (39.4)		38 (42.2)		
**Housework**					0.626^b^
Yes	50 (54.3)		51 (48.0)		
No	42 (45.7)		37 (42.0)		

* P<0.05 is considered significant.

** SMBG: Self-monitoring blood glucose levels.

a P values calculated by Student t-test.

b P values calculated by chi-square test.

### Diet-related Characteristics

Twenty-two percent of women with GDM indicated they never visited a dietitian for counseling while 65% reported they visited a dietitian only once or twice during the pregnancy (Table 2). Only 14% of the women with GDM described their nutrition knowledge as “a lot”. The proportions of women with GDM who reported to have “a lot of control” on food shopping, meal planning, and food preparation for their households were 30.9, 30.1, and 41.5%, respectively (Table 2).

**Table 2:** Diet-related characteristics of pregnant women with and without GDM in Al Ain District.

Variable	GDM (n = 94)	Non GDM (n = 90)	
	n (%)	n (%)	*P* Value*
Did you visit a dietitian during this pregnancy for nutrition counseling?			<0.0001^a^
Yes	73 (77.7)	8 (8.9)	
No	21 (22.3)	82 (91.1)	
How many times did you visit a dietitian during this pregnancyfor nutrition advice on GDM?			
None	21 (22.3)	–	
Once	30 (31.9)		
Twice	31 (33.0)		
Three times	7 (7.4)		
Four times or more	5 (5.3)		
How much knowledge do you think you have about diet and GDM?			
A lot of knowledge	13 (14.0)	–	
Some knowledge	45 (48.4)		
Little knowledge	35 (37.6)		
How much of a control do you have over your household meal planning?			0.23^a^
A lot of control	28 (30.1)	30 (33.3)	
Some control	43 (46.2)	31 (34.4)	
Little/No control	22 (23.7)	29 (32.2)	
How much of a control do you have over your household food shopping?			0.055^a^
A lot of control	29 (30.9)	38 (42.2)	
Some control	50 (53.2)	32 (35.6)	
Little/No control	15 (16.0)	20 (22.2)	
How much of a control do you have over your household food preparation?			0.667^a^
A lot of control	39 (41.5)	35 (38.9)	
Some control	40 (42.6)	36 (40.0)	
Little/No Control	15 (16.)	19 (21.1)	

* P<0.05 is considered significant.

a P values calculated by chi-square test.

### Nutrition Knowledge

The list of the 14 food items used to assess carbohydrate food knowledge is shown in Table 3. The mean carbohydrate-food knowledge score was higher among women with GDM but not significantly different from that of the women without GDM: 8.6±2.2 versus 8.1±2.1, respectively (out of a maximum possible score of 14) (Table 3). This lack of significant difference in knowledge scores between the two groups was also obtained after adjusting the knowledge scores of pregnant women with and without GDM for educational level, nationality, age, parity, and number of visits to a dietitian (8.7±2.1 vs. 8.1±2.1, respectively, P = 0.112). On the other hand, a significantly higher proportion of women with GDM correctly identified rice (86.2% vs. 73.0%, respectively, P = 0.027) and white bread (90.4% vs. 70.8%, respectively, P = 0.001) as food items that can raise blood glucose levels compared with women without GDM. Women with GDM had the least knowledge about three food items that have the potential to increase blood glucose levels (whole wheat bread, low fat milk, and unsweetened fruit juice) as shown in Table 3. In contrast, most women with GDM knew that white bread and whole fat milk can increase blood glucose levels. Also, the majority of women with GDM (93.6%) were able to correctly identify green leafy vegetables as having little effect on blood glucose levels.

**Table 3:** Carbohydrate-containing food knowledge of GDM and non GDM women in Al-Ain District.

Characteristic	GDM (n = 94)		Non GDM (n = 89)		
	Correct responsen (%)	Mean ± SD	Correct responsen (%)	Mean ± SD	*P* Value*
Knowledge Score		8.6±2.2		8.1±2.1	0.112^a^
Knowledge Score**		8.7±2.1		8.1±2.1	0.112^a^
Low fat milk	16 (17)		9 (10.1)		0.165^b^
Honey	79 (84)		72 (80.9)		0.475^b^
White bread	85 (90.4)		63 (70.8)		**0.001** b
Pasta	79 (84)		73 (82.0)		0.716^b^
Rice	81 (86.2)		65 (73.0)		**0.027** b
Vegetable oils	54 (42.6)		44 (49.4)		0.350^b^
Unsweetened fruit juice	32 (34)		24 (27)		0.299^b^
Whole wheat bread	13 (13.8)		10 (11.2)		0.579^b^
Full fat milk	78 (83)		73 (82)		0.865^b^
Fresh fruits	36 (38.3)		30 (33.7)		0.518^b^
Leafy Vegetables	88 (93.6)		80 (89.9)		0.358^b^
Chicken	62 (66)		67 (75.3)		0.167^b^
Meat	42 (44.7)		43 (48.3)		0.622^b^
Potato	80 (85.1)		66 (74.2)		0.065^b^

* P<0.05 is considered significant.

** Adjusted for educational level, nationality, age, parity, and number of visits to dietitian.

a P values calculated by Student t-test.

b P values calculated by chi-square test.

Results from the analysis of the multiple regression showed that the carbohydrate-food knowledge score for women with GDM was significantly associated with participant’s perceived level of knowledge about diet and GDM (P = 0.009) and parity (P = 0.030) (Table 4). On the other hand, carbohydrate food knowledge among women without GDM was significantly associated with parity (P = 0.005) and educational level (P = 0.013) (Table 5).

**Table 4:** Regression estimates and predictors of carbohydrate-food knowledge of pregnant women with GDM (n = 94)^a^.

Model	Unstandardized Coefficients		StandardizedCoefficients	t	P Value*	Correlations		
	B	Std. Error	Beta			Zero-order	Partial	Part
(Constant)	9.400	0.943		9.964	0.000			
How much knowledgedo you have aboutdiet and GDM?	−0.830	0.313	−0.270	−2.655	0.009	−0.285	−0.278	−0.270
Parity	0.722	0.326	0.225	2.210	0.030	0.243	0.234	0.225

Dependent Variable: Knowledge Score (14).

* P<0.05 is considered significant.

a Potential predictors included in the model were: Age, educational level, parity, nationality, previous GDM, and level of participants’ knowledge on diet and GDM.

**Table 5:** Regression estimates and predictors of carbohydrate-food knowledge of pregnant women without GDM (n = 89)^a^.

Model	UnstandardizedCoefficients		StandardizedCoefficients	t	P. Value*	Correlations		
	B	Std. Error	Beta			Zero-order	Partial	Part
(Constant)	4.489	1.224		3.669	0.000			
Parity	0.707	0.247	0.305	2.863	0.005	0.223	0.295	0.290
Educational level	0.628	0.248	0.270	2.536	0.013	0.178	0.264	0.257

Dependent Variable: Knowledge Score (14).

* P<0.05 is considered significant.

a Potential predictors included in the model were: Age, educational level, parity, and nationality.

### Dietary Patterns

Based on the results from the food frequency questions, women with GDM had significantly lower intake of fruits and fruit juices (P = 0.012) and higher consumption of milk and yogurt (P = 0.004) compared to those without GDM (Table 6). When these crude rates were adjusted for possible confounding factors: age, education level, and parity, GDM women with higher education (high school or more) had higher milk intake (P = 0.043). Among the women without GDM, those with higher educational level had higher intake of fruit and fruit juices (P = 0.003).

**Table 6:** Dietary patterns of pregnant women with and without GDM in Al-Ain District.

Variable	GDM (n = 94)		Non GDM (n = 90)	*P* Value*
	n (%)		n (%)	
Milk Group consumption**<1 cup/d	15 (16.0)		24 (26.7)	0.004^a^
1–2 cups/d	39 (41.5)		48 (53.3)	
3 cups	40 (42.6)		18 (20.0)	
Fruit group consumption***				0.012^a^
<1 cup/d	10 (10.6)		10 (11.1)	
1–2 cups/d	52 (55.3)		31 (34.4)	
>2 cups/d	32 (34.0)		49 (54.4)	
Vegetables consumption				0.305^a^
<1 cup/d	15 (16.0)		22 (24.4)	
1–2 cups/d	56 (59.6)		51 (56.7)	
>2 cups/d	23 (24.5)		17(18.9)	
Snacks/d				0.605^a^
None	5 (5.3)		6 (6.7)	
1–2 snacks	62 (66.0)		63 (70.8)	
3 or more snacks	27 (28.7)		20 (22.5)	
	**Mean ± SD**	**Mean ± SD**		
Meals/d	2.9±0.4		2.8±0.4	0.753^b^
Snacks/d	2.0±0.9		1.9±1.1	0.760^b^

* P<0.05 is considered significant.

a P values calculated by chi-square test.

b P values calculated by Student t-test.

** P value changed to 0.043 after adjusting for age, educational level, and parity.

*** P value changed to 0.003 after adjusting for age, educational level, and parity.

Nearly forty-three percent of women with GDM consumed at least 3 cups of milk and yogurt compared to 20% among women without GDM during the week preceding the interview. Moreover, 16% of the women with GDM and 24.4% of those without GDM reported eating less than 1 cup of vegetables per day during the week preceding the interview but the results were not significant. There were no significant differences between the groups in the number of meals or snacks consumed.

### Nutrient Intakes

Based on the results obtained from a single 24 hour diet recall, the mean energy of women with GDM was approximately 1849±65 kcal compared to 2054±99 kcal among women without GDM (Table 7). Although the differences were not statistically significant, women with GDM reported lower intakes of energy and number of macronutrients, including carbohydrate, total fat, and saturated fat. On the other hand, a comparative analysis of the macronutrient intakes revealed a significantly higher percent energy from protein among women with GDM (19.5±0.4% vs. 17.4±0.8, respectively, P = 0.021). There were no significant differences between the two groups with respect to the intakes of any of the micronutrients examined (Table 7). When we adjusted for age, parity, and educational level, using multivariate regression analysis, there were no significant differences in the mean energy and nutrient intakes of pregnant women with and without GDM except for percent energy from protein (P = 0.019).

**Table 7:** Mean energy and nutrient intakes of pregnant women with and without GDM in Al-Ain District as assessed by a single 24 hour diet recall^a^.

Nutrient	GDM (n = 93)	Non GDM (n = 89)	
	Mean ± SE	Mean ± SE	*P* Value*
Energy (Kcal/d)	1849±65.3	2054±99.3	0.084
Carbohydrates			
g/d	252.36±11.20	283.90±14.06	0.080
% Energy	54.16±1.1	55.61±1.00	0.334
Protein			
g/d	87±2.7	85±4.6	0.72
% Energy**	19.5±0.4	17.4±0.8	**0.021**
Total Fat			
g/d	55±3.2	64±4.0	0.068
% Energy	26.4±1.0	26.7±0.8	0.632
Saturated fat			
g/d	19±1.2	22±1.5	0.135
% Energy	9.0±0.5	9.3±0.4	0.654
Calcium (mg/d)	53±46.1	810±36.9	0.079
Iron (mg/d)	13±0.4	15±0.7	0.066
Total Fiber (g/d)	16±1.0	18±1.2	0.169
Cholesterol (mg/d)	286±18.2	304±18.0	0.476

* P<0.05 is considered significant.

a P values calculated by Student t-test.

** P value changed to 0.019 after adjusting for age, educational level, and parity.

## Discussion

Consistent with research conducted elsewhere, women with GDM in this study had a number of known risk factors for GDM [47]. Carbohydrate (CHO) is the macronutrient that has the greatest impact on postprandial blood glucose response. Thus, moderation of carbohydrate intake is crucial in achieving glycemic control among women with GDM [48]. Food items that contain greater than 5g of carbohydrate per serving (such as fruits, milk, grain products, starchy vegetables, and concentrated sweets) have the potential to increase blood glucose levels [42]. In this comparative study of diet and carbohydrate food knowledge of multi-ethnic pregnant women in the UAE, there were no significant differences in the mean knowledge score of food sources of carbohydrate between women with GDM and that of pregnant women without GDM. This was not surprising since only a small proportion (14%) of women with GDM, rated their level of knowledge relating to the dietary management of GDM as “a lot”. Moreover, 22% of women with GDM did not visit a dietitian for nutrition counseling and 65% of them have visited a dietitian only once or twice during their pregnancy despite the fact that medical nutrition therapy practice guidelines recommend a minimum of 3 visits with a dietitian [16]. The low attendance may be due to limited access to dietitians in prenatal clinics. It is also possible that the women did not attend the dietitian visits due to a low awareness about the importance of regular nutrition counseling for effective gestational diabetes management. Given the role of carbohydrate-containing foods on postprandial blood glucose levels, a much higher knowledge than 61% would be desirable for this sample of women with GDM majority of whom were managed with diet and physical activity to facilitate food choices for improved blood glucose levels. A previous study conducted among adults with diabetes in the UAE considered 60.9% as low diabetes knowledge score [49].

Despite the continued efforts by health authorities in the UAE to provide pregnant women high quality healthcare, the nutrition knowledge level among women with GDM this study is alarming. For example, although unsweetened fruit juice contains carbohydrates, more than half of the women with GDM did not think that it could increase their blood glucose levels because it has no added sugar. Moreover, 86.2% of the surveyed GDM women incorrectly thought that compared with white/refined bread, ‘whole wheat’ bread would not affect their blood glucose levels. Similarly, a large proportion of GDM women stated that the whole milk would increase their blood glucose levels compared to low fat milk (83 vs. 17%, respectively). A study conducted in Australia found low CHO food knowledge such as knowledge about food substitutions among multi-ethnic ethnic women. On the other hand, Knowledge about normal blood sugar levels, hypoglycemia and general knowledge about diabetes was high across the different ethnic groups [31].

Although nutrition behaviors are influenced by many factors including economic, educational level, cultural factors, and food availability, nutrition knowledge is an important determinant of the individual’s food choices and dietary behaviors. A study that evaluated the relationship of dietary knowledge and dietary behaviors in rural African American adults found that individuals with knowledge of the effects of fat intake on heart disease were more likely to consume low-fat dairy products [50]. Another study evaluating postpartum weight loss found a greater weight loss among white Hispanic women with higher nutrition knowledge scores compared to those who had lower scores [51].

A dietary pattern that includes carbohydrate from fruits, vegetables, whole grains, legumes, and low-fat milk is recommended for good health among persons with diabetes [10]. In this comparative study of diet of pregnant women with and without GDM, we did not find significant differences in energy and nutrient intakes among women with GDM and those without GDM with the exception of the percent energy from protein. Moreover, despite the recommendations that pregnant women should consume adequate amounts of nutrient –dense foods (including fruits, vegetables, whole grains, and low fat milk) to maintain their own and fetal health [10], our study revealed that 16% of women with GDM consumed less than 1 cup of vegetables per day during the week preceding the interview. Consumption of the DASH eating pattern in pregnant women with GDM have been shown to have to favorably influence the metabolic profiles (including fasting plasma glucose and serum insulin levels) and biomarkers of oxidative stress [52]. Furthermore, specific recommendations for women with GDM is to follow meal plans with carbohydrate intake distributed throughout the day in 3 small to moderate sized meals and 2–3 snacks [30], [53]. The results of our study also showed that more than a quarter of women with GDM (29%) consumed less than 2 snacks per day. This highlights the need for dietitians to emphasize the importance of the daily food intake distribution when counseling women with GDM.

Although large scale studies are still, not conclusive [14], women with GDM may benefit from following a low glycemic index (GI) meal pattern [24], [25], [27], [28]. Moreover, low GI foods may provide additional glycemic control benefits over when total carbohydrate of the meal is considered alone [10]. Such benefits for women with GDM may include lower maternal fasting glycemia and lighter infant birth weights [12]. Therefore, dietitians may consider encouraging women with GDM to choose nutrient-dense lower GI foods, such as legumes, whole grains bread, and oatmeal.

Early initiation and frequent prenatal medical nutrition visits have been consistently shown to improve both maternal and infant outcomes [54], [55]. Dietitians can assist women with gestational diabetes by developing individualized meal plans, as well as implementing and evaluating the care plan through a series of visits. In view of the fact that the UAE has one of the highest prevalence of DM in the world [32], the findings of this study highlight the urgency for health authorities to expand nutrition counseling services to prenatal clinics. Moreover, the treating doctors should expedite referral of pregnant women with GDM to dietitians for nutrition counseling.

### Strengths and Limitations

A major limitation of this study was that it involved a small sample of pregnant women with and without GDM attending 3 hospitals, thus limiting generalization of the results to elsewhere. Another limitation was due to the cross-sectional design used in this study, maternal and fetal outcomes in relation to nutrition knowledge were not assessed. Furthermore, the nutrition knowledge questionnaire used in this study has not been previously validated with women with GDM. However, it was pre-tested with a sub-sample of pregnant women with similar background of the study participants and was previously used for Emirati and Omani adults with type 2 diabetes. On the other hand, an important strength of the study is that for the first time this study provides an important insight into the nutrition knowledge level of women with GDM in the UAE. It also underscores the need to refer pregnant women with GDM to dietitians for nutrition counseling. In conclusion, the results of this study indicate a general lack of carbohydrate-related knowledge among women with GDM and energy and nutrient intakes which are similar to those of pregnant women without GDM.

### Implications for Practice

Health-promoting lifestyle behaviors adopted during pregnancy may promote long-term healthy lifestyle changes. Women with gestational diabetes may be more motivated to seek medical and nutrition advice to achieve improved pregnancy outcomes and have been shown to generally adhere to dietary advice [47]. The findings from this study may be useful to aid the development of nutrition education opportunities for women with GDM in the UAE. Results of this study highlight the urgent need for nutrition counseling and early referral to dietitians among women with GDM. Nutrition practice guidelines for management of gestational diabetes [16] recommend early initiation of the nutrition counseling by a dietitian (within one week of diagnosis). Furthermore, dietitians should discuss with women GDM food sources of carbohydrate and daily carbohydrate intake distribution during nutrition counseling. Such strategies have the potential to improve pregnancy outcomes and decrease future health risks given their markedly increased risks for type 2 DM in future. Finally, the study has implications for the need to assess the knowledge of carbohydrate containing foods among women with GDM in the UAE and elsewhere.

### Recommendations

Future studies in the UAE should evaluate the impact of nutrition knowledge on glycemic control and other important maternal and fetal outcomes. Moreover, further testing should be conducted of on the questionnaire although it was pre-tested. Finally, future studies should explore reasons contributing to the low attendance of women with GDM for nutrition counseling provided by dietitians and ways to increase the frequency of nutrition counseling visits for women with GDM.
